# Contrasted habitats and individual plasticity drive the fine scale movements of juvenile green turtles in coastal ecosystems

**DOI:** 10.1186/s40462-019-0184-2

**Published:** 2020-01-07

**Authors:** Philippine Chambault, Mayeul Dalleau, Jean-Benoît Nicet, Pascal Mouquet, Katia Ballorain, Claire Jean, Stéphane Ciccione, Jérôme Bourjea

**Affiliations:** 1 UMR MARBEC, IFREMER, CNRS, IRD, University of Montpellier, Avenue Jean Monnet, 34200 Sète, France; 2 Centre d’Etude et de Découverte des Tortues Marines (CEDTM), 6 chemin Dubuisson, Appt. 5, 97436 Saint-Leu, La Réunion France; 3 GIE MAREX, 697 Chemin Surprise, La Fontaine, 97436 Saint Leu, La Réunion France; 4 Université de La Réunion/UMR ESPACE-DEV, Antenne SEAS-OI, 40 Avenue de Soweto, 97410 Saint-Pierre, La Réunion France; 5 Biodiversity French Agency, Mayotte and Glorieuses Marine Nature Parks, 6 chemin Dubuisson, Appt. 5, 97436 Saint-Leu, La Réunion France; 6 Kelonia, l’observatoire des tortues marines, 46 rue du Général de Gaulle, 97436 Saint Leu, La Réunion France

**Keywords:** *Chelonia mydas*, Home range, Satellite tracking, Diel pattern, Tidal cycle

## Abstract

**Background:**

A strong behavioural plasticity is commonly evidenced in the movements of marine megafauna species, and it might be related to an adaptation to local conditions of the habitat. One way to investigate such behavioural plasticity is to satellite track a large number of individuals from contrasting foraging grounds, but despite recent advances in satellite telemetry techniques, such studies are still very limited in sea turtles.

**Methods:**

From 2010 to 2018, 49 juvenile green turtles were satellite tracked from five contrasting feeding grounds located in the South-West Indian Ocean in order to (1) assess the diel patterns in their movements, (2) investigate the inter-individual and inter-site variability, and (3) explore the drivers of their daily movements using both static (habitat type and bathymetry) and dynamic variables (daily and tidal cycles).

**Results:**

Despite similarities observed in four feeding grounds (a diel pattern with a decreased distance to shore and smaller home ranges at night), contrasted habitats (e.g. mangrove, reef flat, fore-reef, terrace) associated with different resources (coral, seagrass, algae) were used in each island.

**Conclusions:**

Juvenile green turtles in the South-West Indian Ocean show different responses to contrasting environmental conditions - both natural (habitat type and tidal cycle) and anthropogenic (urbanised vs. uninhabited island) demonstrating the ability to adapt to modification of habitat.

## Background

During their life, animals spend a considerable amount of time on the move [1]. The purposes of movement can vary between species and the various stages of the life cycle, for example long-distance migration to daily foraging movements. Among the multiple causes of daily movements, the search for food and predator avoidance have been largely documented in terrestrial animals [2–6]. For instance, elk move to protective cover of wooded areas when wolves are present [2]. Similarly, Courbin et al. [7] have shown that zebras exhibit a diel migration strategy associated with a particular habitat (vegetation cover at night) to adjust their behaviour to lions’ presence, and therefore reduce the encounter rate with their predator. Diel migrations in the marine realm have also been documented in a large range of marine species, from plankton [8, 9], seals [10, 11], cetaceans [12, 13], fish [14] to sea turtles [15–19].

Using satellite telemetry, such day-night differences have been recently observed in sea turtles. The tracking of loggerhead turtles suggested that these diel patterns might be driven by differences in resource availability (e.g. food vs. nocturnal refuges), competition or exploratory movements [20]. Similarly, sub-adult hawksbill turtles tracked along Florida exhibited day-night patterns, using restricted home ranges at night, likely as refuges [21]. Like their conspecific, a recent study showed a diel pattern in adult green turtles movements as they reduced their activity and home range size at night [15]. Given that sea turtles rely on visual cues to forage and detect predators [22], this nocturnal behaviour is likely associated with resting and/or a predator avoidance strategy, whereas larger home ranges with higher activity levels during day-time may correspond to a foraging activity [15, 23, 24].

Although some behavioural similarities can be observed between individuals from the same species, a wide variation in plasticity responses is commonly evidenced in sea turtles [25–32], and is revealed by contrasting diet [33–35], diving behaviour [25, 26], spatial dynamics [20, 28, 31] or habitat used [25, 36]. For example, adult female green, loggerhead and leatherback turtles explore different habitats during their post-nesting migration, using both neritic and oceanic environments [27, 31, 37]. Similarly, juvenile green turtles from the Atlantic spread in different directions to reach distinct foraging grounds [32]. The reasons for such a plasticity are still unclear, but may be related to the individual’s personality [38], a genetic diversity [32] or an adaptation to local conditions of the habitat [25].

To investigate the behavioural plasticity of sea turtles, this project satellite tracked a large number of juvenile green turtles (*n* = 49) from contrasting foraging grounds located in the South-West Indian Ocean (SWIO). The feeding grounds differed in terms of environmental conditions, e.g. bathymetry, bottom substrate, tidal cycle. The green turtle of the SWIO occupies a large geographical range, including important nesting sites on isolated French territories (Europa, Mayotte, Tromelin and Grande Glorieuse) [39–44], together with some foraging grounds used by both adults and juveniles [45, 46]. The present study aimed at (1) assessing the diel patterns in their movements in contrasting environments, (2) investigating the inter-individual and inter-site variability, and (3) exploring the drivers of their daily movements using both static (seafloor habitat and bathymetry) and dynamic variables (daily and tidal cycles). (i) It was expected that juvenile green turtles would exhibit day-night patterns, using restricted home ranges at night; (ii) individual variations in plasticity is also expected with turtles from the same island selecting different habitats; (iii) it was assumed that a strong inter-site variability would be found, with different habitat features used on each study site. It was felt that the fine-scale mapping of the seafloor habitats of each study site would help in understanding how this species may adapt its behaviour in response to a variety of local conditions, both natural (e.g. mangrove, lagoon) and anthropogenic (e.g. urbanised vs. uninhabited island).

## Methods

### Study areas

**Fig. 1:**
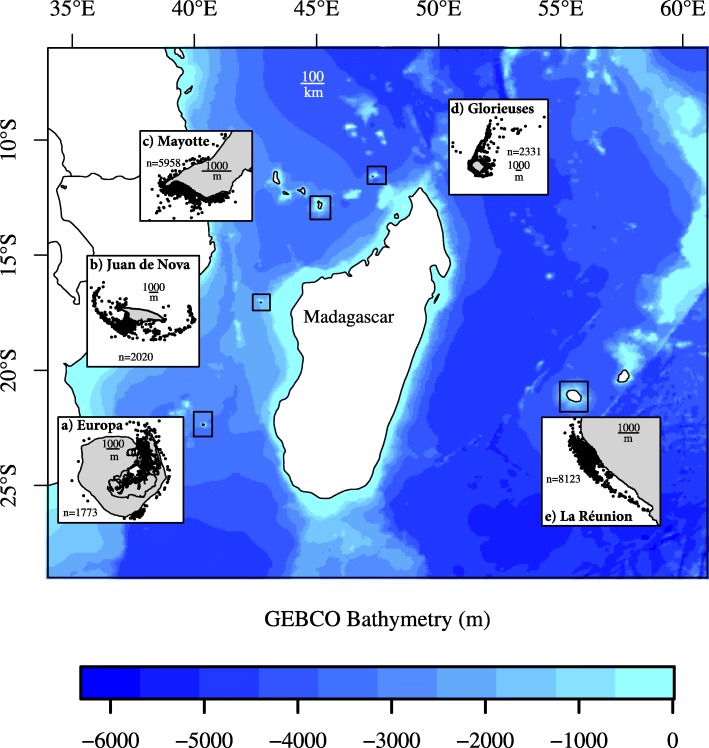
Map of the study area including the five tagging sites: (**a** ) Europa, (**b**) Juan de Nova, (**c**) Mayotte, (**d**) Glorieuses and (**e**) La Reunion. The GPS locations of all tracked turtles are illustrated with the black dots in each box

The large study region spreads from 40 to 55°E and from 11 to 22°S, and is located in scattered French overseas territories of the South-West Indian Ocean, including three French Scattered Islands (Europa, Glorieuses and Juan de Nova), and two Departments (Mayotte and La Reunion) – See Fig. 1. The five study sites differ in terms of anthropogenic pressure as Mayotte and La Reunion are inhabited islands with a developed tourist activity, whereas the three remaining islands (Europa, Glorieuses and Juan de Nova) are uninhabited areas being only a temporary home to French military personnel and scientists.

### Tag deployment

Between 2010 and 2018, 49 juvenile green turtles were caught and satellite tagged in Europa (*n* = 11), Glorieuses (*n* = 10), Juan de Nova (*n* = 9), Mayotte (*n* = 9) and La Reunion (*n* = 10) – See Fig. 1. In-water turtles were captured in shallow waters by rodeo from a boat or by hand (when animals were resting in pools) [47], or scuba diving in deep waters. Once captured, standard morphometric data were recorded for each individual. The curved carapace length (CCL) was measured from the anterior point at midline (nuchal scute) to the posterior notch at midline between the supracaudals [48], and body mass was taken using an electronic dynamometer. Each turtle was photo-identified according to the method developed by Jean et al. (2010) [49]. Argos-Fastloc GPS tags (Wildlife Computers Redmond, WA, USA) that provide Fastloc-GPS data relayed via the Argos satellite system (http://www.argos-system.org/) were then fixed on each juvenile green turtle. In order to increase the number of positions recorded, the Fastloc GPS tags were programmed to record GPS locations at a sampling interval set at 30 min.

### Data pre-filtering

Due to the restricted dispersal pattern commonly observed in juvenile green turtles in their coastal habitats and the large uncertainties associated with Argos locations, only Fastloc locations were retained for the analysis to improve the quality of the results and provide reliable kernel estimates [50]. The Fastloc-GPS data were filtered to reduce measurement errors by removing locations with residuals values above 35 [51] and locations recorded by less than five satellites [51]. We restricted our dataset to positions associated with a travel speed lower than 5 km.h^− 1^ [29]. Finally, remaining positions located on land were discarded, representing between 2 and 17% of the dataset. To investigate diel movement patterns, locations were assigned as either day-time or night-time (using the *suncalc* package in R) that provides precise local time of sunrise and sunset.

### Home range analysis

To investigate the residency pattern of the turtles, locate the high-use areas and estimate their home range size, a kernel utilisation density approach was used [52]. The use of the reference bandwidth parameter *h*_*ref*_ as smoothing parameter generally results in over-smoothing the data [53]. Conversely, a bandwidth that minimises the least-square cross validation score (*h*_*lscv*_) often under-smoothes location data [54]. To prevent over and under-smoothing, we therefore used a visual ad hoc approach previously applied to terrestrial animals [55, 56]. We first calculated the reference bandwidth parameter *h*_*ref*_ for each turtle. Then, *h*_*ref*_ was sequentially reduced in 0.10 increment (0.9 *h*_*ref*_, 0.8 *h*_*ref*_, 0.7 *h*_*ref*_, …) until 0.1 *h*_*ref*_, and the most appropriate smoothing parameter was chosen visually by comparing the kernel density to the original location data [53]. Using this method, one kernel density was estimated for each individual and each day phase (day vs. night). The areas covered by the diurnal and nocturnal home ranges (50% contour, [52]) were then estimated for each individual using the *adehabitatHR* package. Individuals tracked for less than 10 days were discarded from the kernel analysis.

Kernel density estimates are known to be sensitive to sampling regime (i.e. tracking duration and number of locations recorded) [57]. To address these potential bias and allow a comparison of kernel areas across individuals, we performed two sensitivity analyses to assess the potential influence of (i) the tracking duration and (ii) the number of locations on kernel estimates. Firstly, kernel areas (diurnal and nocturnal, separately) were calculated individually for different timeframes, i.e. every 30 d from 30 to 630 d. Secondly, kernel areas (diurnal and nocturnal, separately) were calculated individually for different numbers of locations selected randomly over the entire tracking length of each individual, i.e. every 10, 20, 50, 100, 150, 200, 350, 500, 700, 1000, 1500, 2000 and 2750 locations. The calculated areas were then compared using correlation matrices for each study site and each day phase.

### Environmental variables

Four environmental variables (both static and dynamic) were used to investigate the drivers of the turtles’ coastal movements:

1. *Influence of depth*: the fine-scale bathymetry (spatial resolution from 1 m to 5 m, up to a depth of 40 m) was first extracted at each turtle position using the *Litto3D* product provided by the SHOM (Service Hydrographique et Océanographique de la Marine, http://www.shom.fr/les-activites/projets/modele-numerique-terre-mer/couverture/ocean-indien/).
2. *Influence of the distance to shore*: using maps of each island shoreline, the shortest distance between each turtle location and the coastline was also calculated.
3. *Influence of the seafloor habitats*: maps of the seafloor habitats available (characterised by their geomorphology, dominant benthic communities, roughness and exposition) were generated for all sites except Juan de Nova (data unavailable) [58]. A total of 11 habitats was identified on the four sites (see Additional file 6: Figure S6):
  Lagoonal terrace (hereafter called “Terrace”),
  Mangrove,
  Unexposed fore-reef (“Fore-reef”),
  Exposed fore-reef (“E. fore-reef”),
  Exposed fore-reef with high complexity (“C. fore-reef”),
  Unexposed reef flat (“Reef flat”),
  Exposed reef flat (“E. reef flat”),
  Seagrass,
  Fore-reef and reef patch of terrace (“Reef patch”),
  Blind pass (“Pass”),
  And Land.
4. *Influence of the tides*: tidal cycles (e.g. tidal height) were calculated for every 10 min at the location of each study site from the SHOM database. Then a sea height value was attributed to each turtle’s location according to the corresponding tidal time previously extracted at the study site.

### Habitat selection analysis

Habitat use and habitat selection were assessed by compositional analysis using the *adehabitatHS* package [59]. To take into account the potential diel pattern, the analysis was conducted separately for the diurnal and nocturnal habitats. The habitat available was defined as the habitat located within the Maximum Convex Polygon (95% MCP) of all turtles of each study site, and it was calculated for day and night positions using the *adehabitatHR* package. The habitat used corresponded to the individual 50% kernel contours calculated during day and night for each turtle (the individuals kept for the kernel analysis, *n* = 48). By quantifying the ratio of the used against the available habitat [60], selection ratios were computed for each site and each time of the day to assess the habitat affinities according to day-time and night-time (e.g. feeding during the day vs. resting at night).

To investigate the inter-site and inter-individual variabilities, several Multiple Analysis of Variance (MANOVA) were tested using R by taking different dependent (DVs) and independent variables (IVs). (1) To test if turtles from the same island behaved differently, we tested for each site, the turtle ID as IV, and 5 DVs: bathymetry, sea height, habitat type, distance to shore and phase of the day. (2) To test if the turtles from different islands showed distinct behaviours, we performed a MANOVA with the site as IV and four DVs: bathymetry, sea height, distance to shore and phase of the day. (3) To test behavioural differences between day and night, we run MANOVA for each site, taking the phase of the day as IV and four DVs: bathymetry, sea height, habitat type and distance to shore. (4) Finally, to investigate which environmental predictor influenced the most the behaviour of the turtles, we performed MANOVAs for each site, taking alternatively the habitat type, bathymetry, sea height and distance to shore as IVs, and the geographical coordinates as DVs.

### Habitat modelling

To investigate the diel pattern and the effect of tides on turtle movements, we constructed a series of Generalised Additive Mixed Models (GAMMs) using the *mgcv* package in R [61]. The *distance to shoreline* was used as a response variable and a *scat* distribution was applied to the models to deal with the heavy tailed data. One model was run for each of the five study sites. Turtle *ID* was used as a random effect and therefore added as an explanatory variable. To reduce autocorrelation, the dataset was subsampled every 2 to 6 locations, and autocorrelation was then tested using the *acf* function in R. Two environmental predictors were used: *time of the day* and *tidal height*. Due to its circular distribution, a cyclic cubic regression spline (type “*cc*” in *mgcv* package) was used for the response variable. The predictors were first tested for collinearity using the Variance Inflation Factor (below three). Models with all possible combinations were then computed, and the models were compared based on the Akaike Information Criterion (AIC). The model residuals (QQ-plot and histogram) were then checked for normality to validate the most parsimonious model. When necessary, the response variable was log-transformed to make residuals homogeneous.

## Results

### General tracking data

After filtering, a total of 49 juvenile green turtles were satellite tracked in Europa (*n* = 11), Glorieuses (*n* = 10), Juan de Nova (*n* = 9), Mayotte (*n* = 9) and La Reunion (*n* = 10), representing a large dataset of 20,277 GPS locations used in the analysis (Additional file 7: Table S1). The number of GPS locations per individual varied between 13 (#112120 in Europa) and 2723 (#32899c in La Reunion). The turtles measured on average (±SD) 59.8 ± 8.1 cm CCL and weighed 25.8 ± 10.8 kg. The tracking duration was on average 136 ± 104 days (range: 6–627 d). Among the 49 tracked individuals, only two turtles tagged in Europa (#32874b and # 32905b) left the island to reach the West coast of Madagascar whereas the 47 other turtles remained close to their release point (Additional file 1: Figure S1). The two turtles that departed remained in Europa waters for 92 (#32874b) and 68 days (# 32905b) respectively before leaving the island.

### Home range

**Fig. 2:**
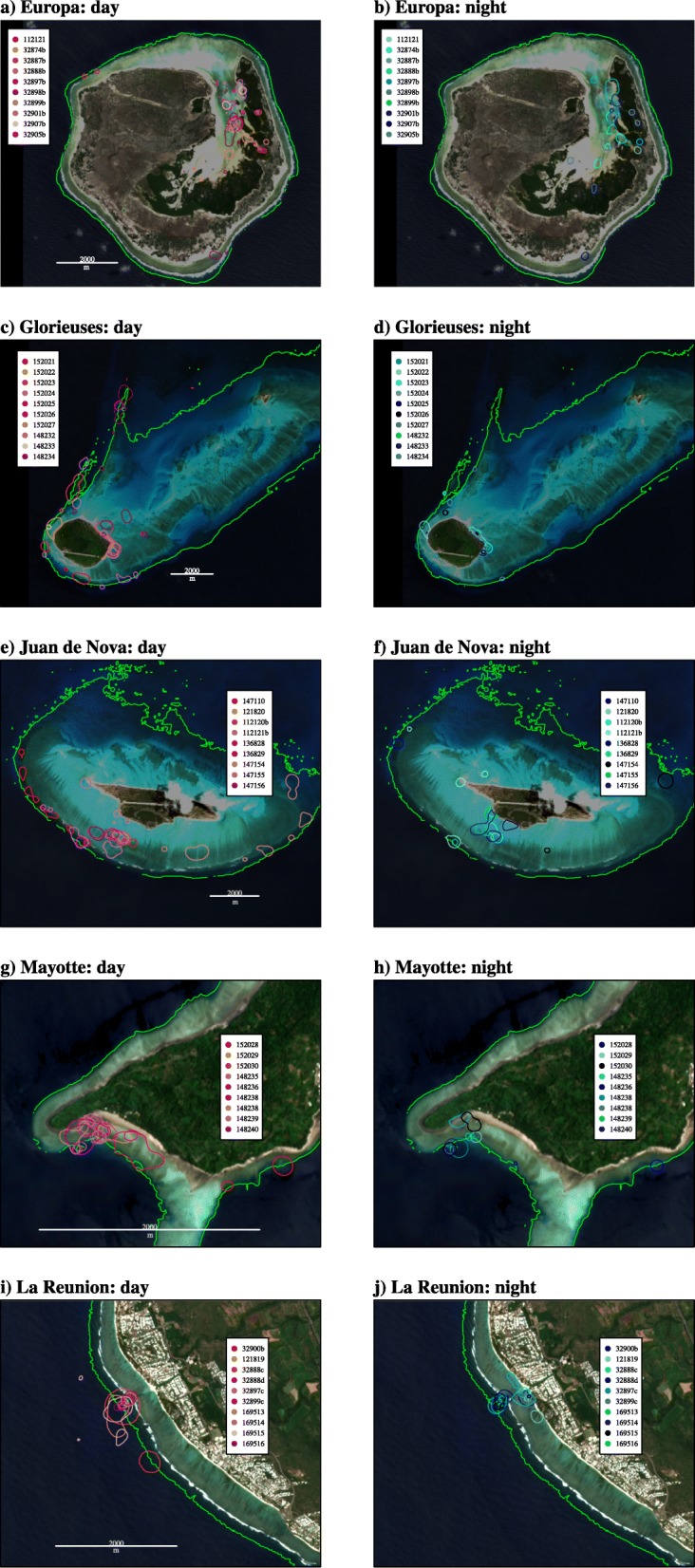
Individual kernel densities (50% contours) during day and night for (**a**, **b**) Europa, (**c**, **d**) Glorieuses, (**e**, **f**) Juan de Nova, (**g**, **h**) Mayotte and (**i**, **j**) La Reunion. The green lines in each plot refer to the 10 m isobaths

Among the 49 individuals tracked, 1 turtle was discarded from the kernel analysis due to a short tracking duration (< 20 d, #112120). The remaining turtles (*n* = 48) dispersed in shallow waters at all sites, rarely exceeding the 10 m isobaths (Fig. 2). Turtles dispersed much more in Glorieuses and Juan de Nova than in the three other sites. All the individuals except two, remained inside Europa’s mangrove. In Mayotte and La Reunion, the turtles also showed limited displacements. Both sensitivity analyses showed very limited influence on either the tracking length (Additional file 2: Figure S2), or the number of locations on the kernel estimation for all sites (Additional file 3: Figure S3). Indeed, the correlation matrices calculated for day and night indicated a strong correlation between the kernel areas of different tracking lengths (mean correlation coefficient ± SD: 0.96 ± 0.5) and number of locations (mean correlation coefficient ± SD: 0.93 ± 0.09).

Numerous high-use areas were identified showing a small proportion of overlap between individuals (Fig. 2). Except in Europa and in the East of Glorieuses (main island, Fig. 2a, b, c, d), where most of the high-use areas overlapped between day and night, a diel pattern was observed in the movements of the majority of the individuals in all study sites. In Mayotte, most of the turtles remained between the coastline and the 10 m isobaths, and in shallower waters during the day (Fig. 2g, h). The opposite pattern was observed in La Reunion, where the turtles concentrated their activity in deeper waters during the day, and remained in shallower waters (< 10 m deep) closer to shore at night (Fig. 2i, j).

**Fig. 3:**
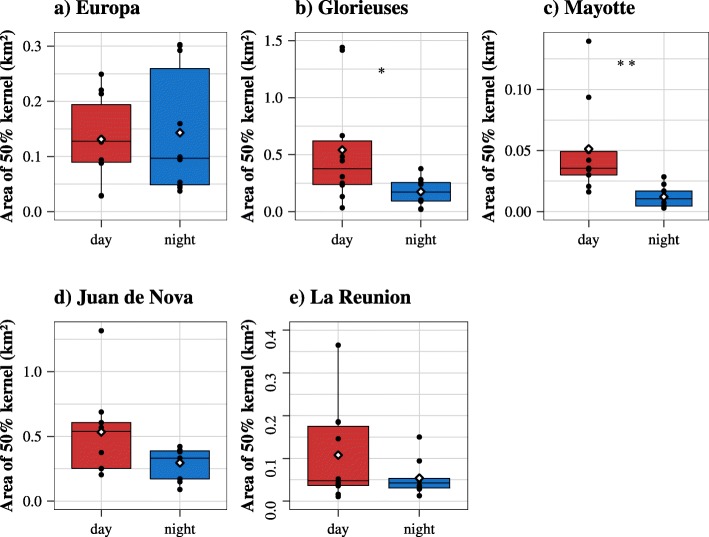
Box plots of the 50% kernel areas (in km^2^) according to the time of the day for (**a**) Europa, (**b**) Glorieuses, (**c**) Mayotte, (**d**) Juan de Nova and (**e**) La Reunion. The black dots refer to the means of each individual and the white diamonds to the means of each boxplot. The stars stand for the *p*-values, i.e. *p* < 0.001 (***), *p* < 0.01 (**), *p* < 0.05 (*)

The home ranges were relatively small for all sites (mean: 0.21 ± 0.27 km^2^, Fig. 3), but some differences were observed between sites and individuals. Except in Europa, the area covered by the 50% kernel contour was smaller at night. However, it was significant for Glorieuses (Wilcoxon test, *p* < 0.05) and Mayotte (Wilcoxon test, *p* < 0.01), whereas no significant difference was observed in Juan de Nova (Wilcoxon test, *p* = 0.0976), Europa (Wilcoxon test, *p* = 0.3750) and La Reunion (Wilcoxon test, *p* = 0.0839). A strong inter-individual plasticity was observed when looking at the areas used by the turtles during day and night (Fig. 3). For example, in Juan de Nova, the individual kernel areas ranged between 0.2 (#121820) and 1.3 km^2^ (#147154) – See Fig. 3d.

### Distance to shore

**Fig. 4:**
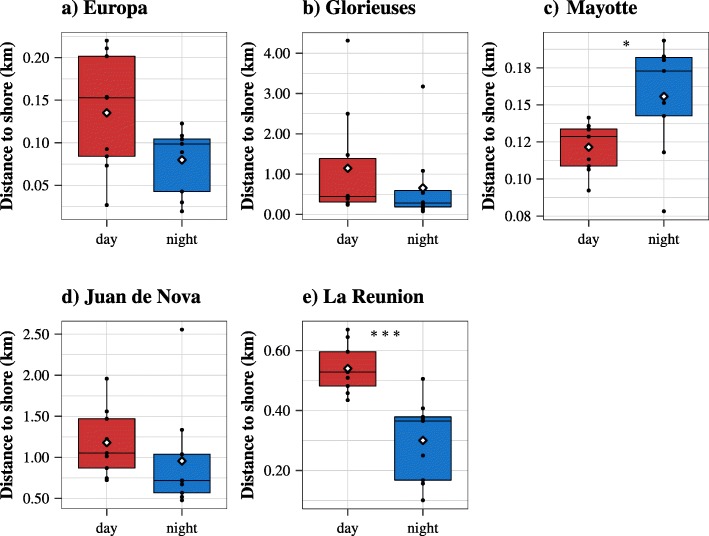
Box plots of the distance to shore (in km) according to the time of the day for (**a**) Europa, (**b**) Glorieuses, (**c**) Mayotte, (**d**) Juan de Nova and (**e**) La Reunion. The black dots refer to the means of each individual and the white diamonds to the means of each boxplot. The stars stand for the *p*-values, i.e. *p* < 0.001 (***), *p* < 0.01 (**), *p* < 0.05 (*)

For all sites, a diel pattern was observed in terms of distance to shore. Except for Mayotte, the distance to shore was shorter at night than during day-time (Fig. 4). This difference was significant for Mayotte (Wilcoxon test, V = 8, *p* < 0.05) and La Reunion (Wilcoxon test, V = 55, *p* < 0.005). A strong inter-individual plasticity was observed when looking at the average distance to shore (Additional file 4: Figure S4). For example, in Glorieuses, the average distance to shore calculated for each turtle ranged between 0.24 (#152022) and 4.31 km (#152026) – See Fig. 4b and Additional file 4: Figure S4.

**Fig. 5:**
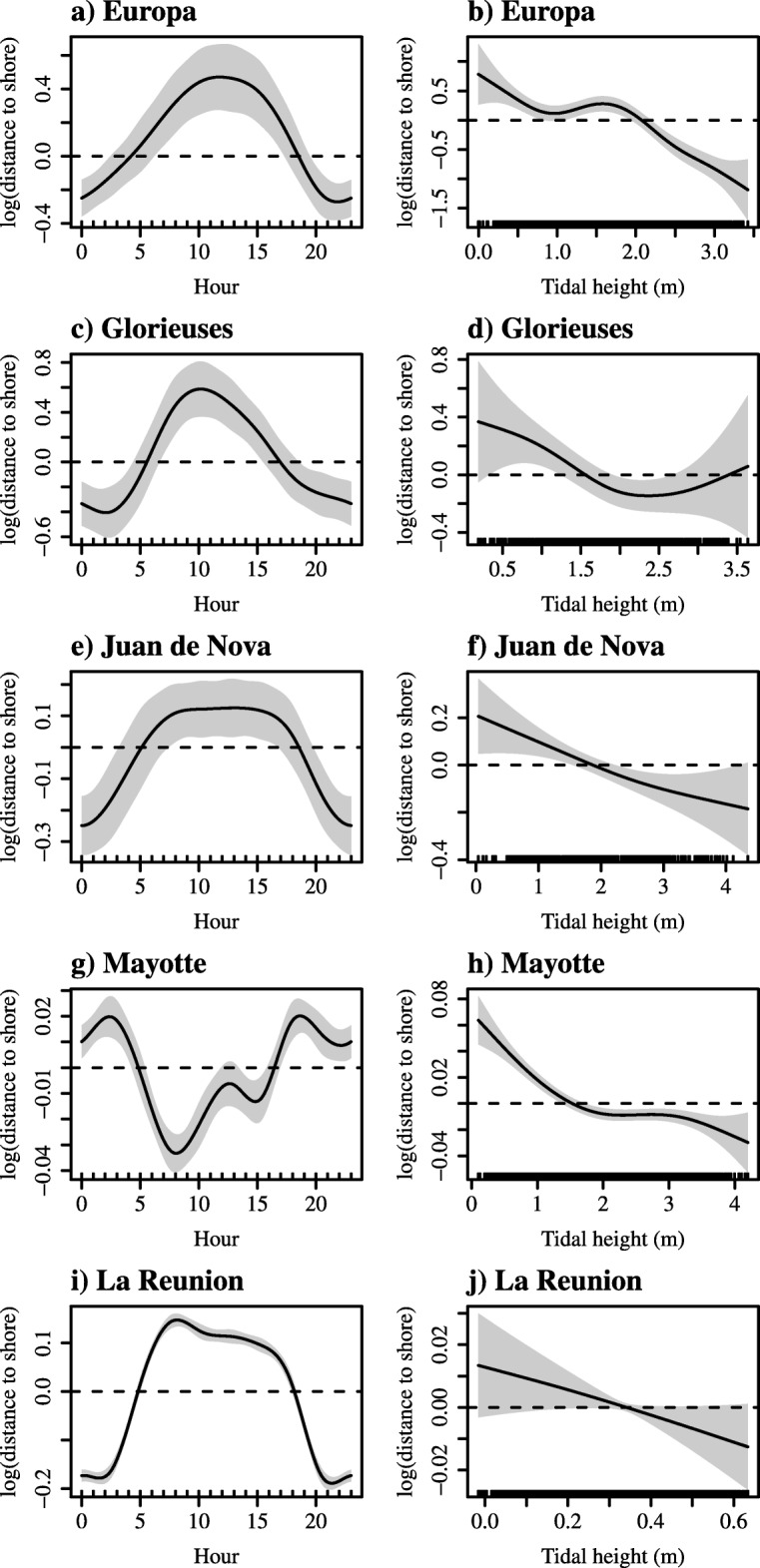
Relationships between the distance to shore and time of the day and sea height obtained from the GAMMs for (**a**, **b**) Europa, (**c**, **d**) Glorieuses, (**e**, **f**) Juan de Nova, (**g**, **h**) Mayotte and (**i**, **j**) La Reunion. The solid black line in each plot is the smooth function estimate and the shaded regions refer to the approximate 95% confidence intervals. The Y-axis represents the response variable (distance to shore) expressed in log scale. The horizontal dotted line indicates no effect of the variable

The results from the GAMMs confirmed the strong relationship between the distance to shore and the time of the day for all sites (Fig. 5a, c, e, g, i). Except in Mayotte, the distance to shore increased during day-time, and decreased at night. Conversely, the turtles were closer to shore during day-time in Mayotte, being however further off the shoreline at noon (Fig. 5g). The explained deviances ranged from 18% in Mayotte to 57% in La Reunion, and the selected models contained both *time of the day* and *tidal height* as explanatory variables. For all sites, distance to shore decreased with increasing tidal height, in relation to tidal cycles. The GAMMs also showed a negative relationship between distance to shore and tidal height (Fig. 5b, d, f, h, j).

### Bathymetry

The bathymetry extracted at the turtles’ locations ranged from 0.5 to 37 m deep (Additional file 5: Figure S5). Turtles mainly used shallow habitats, with mean depths ranging from 1.5 m in Europa to a maximum of 7.5 m in La Reunion. Differences were observed between day and night, with shallower depths used at night for all sites but Mayotte. However, this diel pattern was only significant in Glorieuses (Wilcoxon test, V = 53, *p* < 0.05) and La Reunion (Wilcoxon test, V = 55, *p* < 0.005). Unlike the four other sites, the bathymetry used by the turtles decreased during the day in Mayotte and increased at night (Wilcoxon test, V = 3, *p* < 0.005). A strong inter-individual plasticity was observed when looking at the bathymetry associated with each individual’s location (Additional file 5: Figure S5). For example, in La Reunion, the average bathymetry extracted for each turtle ranged between 2.0 (#169516) and 13.6 m (#169513).

### Habitat selection

Regarding the seafloor habitat available, the habitats are illustrated in Additional file 6: Figure S6. *Seagrass* was common to Glorieuses, Mayotte and La Reunion, *Exposed reef flat* to Europa, Glorieuses and La Reunion, and *Terrace* to Europa, Glorieuses and Mayotte.

**Fig. 6:**
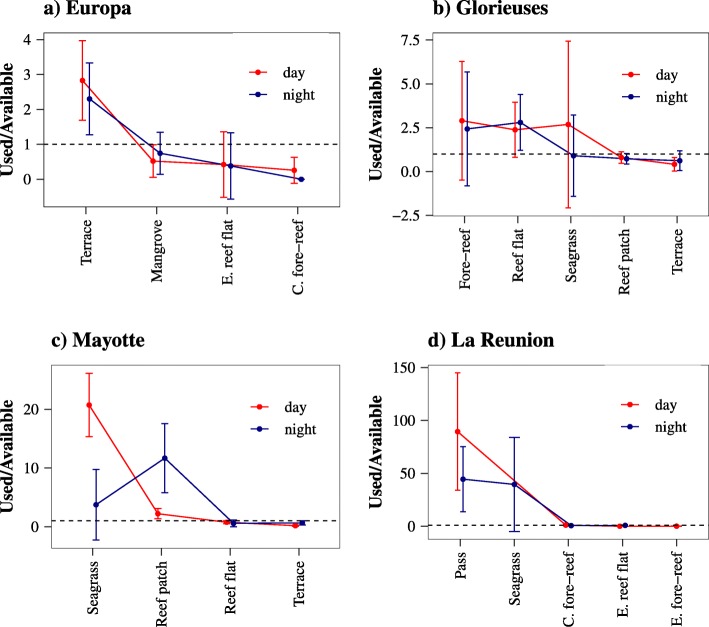
Resource selection ratios (habitat used/habitat available) for (**a**) Europa, (**b**) Glorieuses, (**c**) Mayotte and (**d**) La Reunion according to the time of the day and the habitat type. Selection ratios below 1 mean habitat avoided, vs. above 1: habitat selected. *C. fore-reef* refers to *Fore-reef with high complexity* and *E. reef flat* to *Exposed reef flat*.

In Europa, the home ranges were mainly located on the *Terrace*, which was the most selected habitat, regardless the time of the day (Fig. 6a and Additional file 6: Figure S6a). Only two turtles used the *Exposed reef flat* and the *Complex fore-reef*.

In Glorieuses, individuals used three major habitats (*Fore-reef*, *Reef flat* and *Seagrass*), but no significant difference was observed between day and night (Fig. 6b and Additional file 6: Figure S6b, d). A large inter-individual variability was also observed based on large standard errors (Fig. 6b).

In Mayotte, the dominant habitat selected during day-time was *Seagrass*, whereas the *Reef patch* was preferred at night (Fig. 6c and Additional file 6: Figure S6c). Only two individuals also used the *Seagrass* at night.

In La Reunion, the habitat *Pass* was largely selected during both day and night (Fig. 6d and Additional file 6: Figure S6d). However, the turtles also used the *Seagrass* at night, located closer to shore.

The inter-individual and inter-site variabilities in terms of habitat used were confirmed by the MANOVA analysis. Individuals from the same island (*turtle ID* as IV) selected different habitats (DVs), i.e. bathymetry, sea height, seafloor substrate and distance to shore (MANOVA, *p* < 0.001). Similarly, turtles from different sites (*site* as IV) selected different habitats (DVs, MANOVA, *p* < 0.001). Also, the use of distinct diurnal and nocturnal habitats (*phase of the day* as IV) was evidenced for all sites (*geographic coordinates* as DVs, MANOVA, *p* < 0.001). Finally, except *sea height*, all environmental variables (IVs) had a significant effect on turtle distribution (*geographic coordinates* as DVs) at all sites (MANOVA, *p* < 0.001).

## Discussion

By compiling a large dataset of 49 juvenile green turtles satellite tracked in the South-West Indian Ocean from five contrasting feeding grounds, this study sheds light on the diel patterns movements and inter-individual and inter-site plasticity of this species. The analysis of the turtle locations (*n* = 20,277) in relation to their fine-scale habitat types (seafloor habitat, bathymetry and tidal height) enabled the characterisation of their (i) diurnal and (ii) nocturnal habitats, highlighting a pronounced behavioural plasticity.

### Diurnal habitats

The long tracking duration (mean ± SD: 136 ± 104 days) and the small home ranges (mean ± SD: 0.18 ± 0.25 km^2^) found in this study confirmed the strong site fidelity of juvenile green turtles to their developmental habitats, regardless of the foraging ground. Turtles limited their movements by remaining particularly close to their release positions. These results are in agreement with previous studies conducted in the Caribbean [24, 62–66], Atlantic [17, 67, 68], and to a lesser extent in the Mediterranean Sea [20] and Pacific [69]. Kernel density estimates are known to be sensitive to sampling regime, via the number of locations and tracking duration that vary among individuals [57]. The normal method to give the same weight to all individuals is to average the dataset to daily locations [29]. Such procedure is inappropriate when looking at very fine scale movements (~tens of meters) in relation to habitat features, as it could have generated erroneous positions associated with wrong habitats. The sensitivity analysis performed in this study to test for a series of different tracking lengths confirmed that a nonhomogeneous sampling does not necessarily impact kernel estimations, making comparisons across turtles reliable. Similarly, the second sensitivity analysis conducted on different number of locations supported the comparison of the kernel areas across individuals. For these reasons, these kernel estimates provide a reliable indication of the core activity of diurnal and nocturnal sites used by this species and the associated habitat selected, and such a complete approach for tracking studies when using kernel densities is recommended.

The areas covered by the home ranges differed between day and night, with globally larger home ranges during day-time. Such diel patterns have also been documented in other sea turtle species, including the loggerhead and the hawksbill, and might be partly driven by differences in resource availability (food vs. nocturnal refuges) [20, 21]. In Mayotte, the strong overlap between the diurnal locations of the turtles and seagrass beds confirmed previous results [46], as the turtles exploited the seagrass meadow during the day. In Glorieuses, sparse seagrass patches (occur in some areas of the reef flat) and a large seagrass bed (located several kilometres away from the island) were used by only three individuals. If these individuals do feed on such seagrass species, it suggests a trade-off between the energy gain by consuming seagrass and the energy loss of travelling towards this specific habitat. Such inter-individual variability could also be due to intra-specific competition, as evidence by Dujon et al. [20]. This large and dense seagrass bed is composed of only one species (*Thalassodendron ciliatum* [58, 70]), which is not usually consumed by green turtles [46], suggesting a generalist rather than a specialist behaviour and the use of alternative resources. Immature green turtles could therefore preferentially select seagrass beds when they are sufficiently abundant-accessible, and contain the preferred species; alternatively they would target substitute habitats.

The distance to shore also varied between diurnal and nocturnal habitats, with generally the use of deeper habitats farther from shore during the day. Although three different patterns were observed in Mediterranean loggerhead turtles, most of the tracked individuals in this study also used night-time sites closer to shore [20], likely as refuges from predators. In the foraging ground close to urbanised zones of La Reunion, the turtles favoured the slope located outside the lagoon during the day. Such diel pattern could be a strategy to avoid human disturbance during day-time [71], forcing the turtles to leave the lagoon in response to seaside tourism. While this human avoidance tactic could be reliable in La Reunion due to the intense leisure activity [72–74], this strategy is not adopted in Mayotte, despite significant human activities. The net energy gain induced by feeding on the large seagrass meadow located in the nearshore waters of Mayotte might counterbalance the disturbance caused by tourists. In contrast, the scarcity of such resources inside the lagoon of La Reunion (e.g. small patches of the monospecific seagrass beds *Syringodium* occur, but there is neither algae nor coral) may explain the aggregation of the turtles away from the shore during the day. During day-time, turtles from La Reunion select the habitat *Pass*, likely to transit easily between the outer core and the inner core of the lagoon from diurnal to nocturnal sites, and for cleaning, feeding on corals or resting in caves [75]. A similar pattern was observed in loggerhead turtles tracked in the Mediterranean Sea [20], since some individuals used distinct day and night refuges with minimal overlap.

Unlike the four other foraging grounds, Europa was the only site where no difference in terms of home range size was observed between day and night. The particular geomorphology of the island (i.e. a semi-closed mangrove) providing simultaneously a shelter from predators and food resources might remove the necessity to shift between resting and foraging habitats. The use of overlapping day and night sites might increase feeding efficiency while minimising energy expenditure [76], and this strategy has already been observed in juvenile green turtles in Florida [17] and loggerhead turtles in the Mediterranean Sea [20].

Mangroves are complex ecosystems where juvenile green turtles have been observed feeding on leaves, propagules and fruit [33, 34, 47, 77–79]. Mangroves have been observed serving as nurseries for dolphins Southern of Brazil [80], as a result of an abundance in nutrients, fishes, crustaceans and algae [81, 82], while providing a refuge from predators. Despite mangroves in Europa serving as an alternative food supply, the resource might be less nutritiously and energetically less profitable than in the other sites, leading to slower growth rates [83], and making the turtles leave this feeding ground earlier than might be expected. The departure of two individuals that reached the west coast of Madagascar after spending only 2 to 3 months around the island lends some credence to this hypothesis. The smaller size of the individuals regularly measured in Europa (Bourjea, personal communications) compared to those of Glorieuses also suggests that the habitat found in Europa’s mangrove might be less profitable. The large size and diversity of habitats available in Glorieuses compared to Europa might also explain the strong inter-individual variability and the use of multiple habitats.

### Nocturnal habitats

The turtles tracked in this study used smaller habitats at night in all study sites, which reinforces the hypothesis that sea turtles decrease their activity during night-time [15, 17, 84]. Such behaviour has also been observed in other species such as the loggerhead [20, 85] and the hawksbill [21, 86], suggesting a tactic to reduce predation risk [22], as turtles generally rest close to reef structures where they can find shelter in small caves and under reef ledges [17, 87]. This is probably the case in La Reunion [75], Glorieuses and Mayotte (Ballorain, personal communication), where many juvenile turtles are commonly observed resting in small caves. The question of the impact of predation has lately been addressed as one of the key questions in megafauna movement ecology [88], but remains poorly documented for sea turtles. To confirm if predation risk is a key factor in turtle movements in such islands, it will be necessary to conduct a dedicated study including direct observations of the relationship between sharks and green turtles, with some emphasis on corticosterone measurements (i.e. stress hormone).

In Mayotte, such a pattern was confirmed by the use of the *Reef patch* at night. The lengthy and deep resting dives recorded at night on coral and rocky habitats by adult green turtles in Mayotte [19] suggest a similar behaviour to that adopted by the juveniles in the same foraging ground. Although sea turtles rely mainly on visual cues to feed, some scattered records observed at night on the seagrass beds of La Reunion and Mayotte suggest that they could also feed during night-time, confirming strong behavioural plasticity. Unlike the stable and relatively static seafloor habitats (e.g. reefflat, slope), the dynamic seagrass beds might influence differently across years the habitat selected by the turtles. This is particularly true in La Reunion where the small patches of seagrass located on the reefflat are gradually disappearing over time, explaining why individuals tracked from 2018 (*n* = 4) selected less seagrass at night. Influenced by the moonlight, adult green turtles have already been observed feeding during full moon in Mayotte [19, 89], but no such relationship could be confirmed in this study. Both the spatial and temporal fine-scale behaviour of these individuals needs to be further investigated using time-depth recorders and cameras to develop a better understanding of the feeding activity in relation to the associated habitat.

As with similar studies on green turtles from Mexico and the Chagos Archipelago, nocturnal habitats were mostly located closer to land [15, 18]. The opposite behaviour observed in Mayotte (in deeper waters at night) might be driven by turtle’s buoyancy. Previous studies have demonstrated that long resting dives in sea turtles might be achieved by reaching neutral buoyancy at a certain depth (~ 19 m) both to reduce energy expenditure and perform longer dives [90, 91]. Such behaviour may be adopted by juvenile green turtles in Mayotte.

The tidal cycle might also explain turtle movements, forcing individuals to move away from the shoreline at low tides as some areas might become inaccessible. Given the negative relationship between distance to shore and tidal height, such hypothesis was supported in all sites. During periods of strong tidal coefficient in all islands (except La Reunion which has a small tidal range < 1 m), the area of the available habitat can be considerably reduced. Such occasional phenomena explains the erratic movements of some individuals from Europa that travelled outside the mangrove due to a lack of water in the inner core of the island. Similarly, movements away from shoreline were observed in Glorieuses, Juan de Nova and Mayotte, coinciding with the decreasing sea level induced by the tidal cycle. Remaining in close proximity to the tidal flow could also provide more opportunities to catch prey, especially those trapped due to water movements or benthic animals that emerge at rising tides. During flood tides, the water is generally more turbid, and using these turbid waters could be used by turtles as a tactic to avoid predators.

## Conclusion

Although similarities in terms of movements were observed between the five foraging grounds, it is also worth mentioning the strong inter-site and inter-individual variability. The high degree of plasticity in sea turtles’ movements and home ranges has already been recorded in numerous studies [17, 89, 92–94], but this is the first time such plasticity has been demonstrated by a meta-analysis of juvenile green turtles’ movements tracked from five contrasted sites from the same Regional Management Unit [95]. Inter-individual variability could be attributed to both intrinsic (e.g. level of experience, personality, metabolism rate, competition) and extrinsic factors (e.g. environmental perturbations, resource availability, predation). The contrasted habitats and associated resources observed at the five sites also contribute to this variability, and may reveal some dietary adaptations. The green turtle is known to have an omnivorous diet at this stage, feeding either on animal matter (e.g. cephalopods [96], gelatinous zooplankton [34, 35]), marine angiosperms (seagrass [96–98] or algae [33, 77, 99]). Stable isotope analysis should be conducted in the near future to investigate the diet of these juvenile green turtles at their foraging grounds, which may provide crucial information explaining the variability in their movements and habitat use. Investigating simultaneously the growth rate, the energy and nutrient content of the resources available and the quantities consumed in each habitat could provide an indication of the drivers of this behavioural plasticity.

## Supplementary information

**Additional file 1: Figure S1.** GPS locations on (a) Europa, (b) Glorieuses, (c) Juan de Nova, (d) Mayotte and (e) La Reunion. Red dots refer to release locations. (f) Migratory movements of two individuals that left Europa.

**Additional file 2: Figure S2.** Correlation matrices of the kernel areas tested for different tracking durations during day (left) and night (right) in (a, b) Europa, (c, d) Glorieuses, (e, f) Juan de Nova, (g, h) Mayotte and (i, j) La Reunion. Tracking durations are numbers in red.

**Additional file 3: Figure S3.** Correlation matrices of the kernel areas tested for different number of locations during day (left) and night (right) in (a, b) Europa, (c, d) Glorieuses, (e, f) Juan de Nova, (g, h) Mayotte and (i, j) La Reunion. Locations numbers are numbers in red.

**Additional file 4: Figure S4.** Box plots of the distance to shore extracted at each turtle location showing the inter-individual variability.

**Additional file 5: Figure S5.** Box plots of the bathymetry extracted at each turtle location showing the inter-individual variability.

**Additional file 6: Figure S6.** Maps of the seafloor habitats in (a) Europa, (b) Glorieuses, (c) Mayotte and (d) La Reunion. Habitat available are illustrated by the MCP (dotted black lines) and the individual habitat used by the red (diurnal) and blue (nocturnal) contours.

**Additional file 7: Table S1.** Summary of the data collected from the 49 juvenile green turtles satellite tracked. N refers to the total number of GPS locations retained for the analysis.

## Data Availability

Data sharing not applicable to this article as no datasets were generated or analysed during the current study. If you do not wish to publicly share your data, please write: “Please contact author for data requests.

## Abbreviations

%: Percent
AIC: Akaike Information Criterion
CCL: Curved Carapace Length
d: Days
°E: Degree East
Fig.: Figure
GAM: Generalised Additive Model
GPS: Global Positioning System
h: Hours
*h*_*lscv*_: Least-square cross validation smoothing parameter
*h*_*ref*_: Reference bandwidth smoothing parameter
ID: Individual number
km: Kilometres
m: Metres
MCP: Maximum Convex Polygon
min: Minutes
n: Sample size
°S: Degree South
SD: Standard deviation
SHOM: Service hydrographique et océanographique de la marine
SI: Supplementary information
