# Supplementary figures and images for: Contrasted habitats and individual plasticity drive the fine scale movements of juvenile green turtles in coastal ecosystems
Source: Mov Ecol. 2020 Jan 7;8:1. doi: 10.1186/s40462-019-0184-2 (PMC6947949; doi:10.1186/s40462-019-0184-2)

**a) Europa**

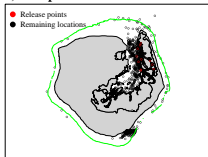

**b) Glorieuses**

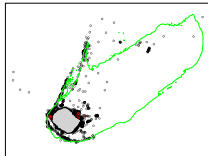

**c) Juan de Nova**

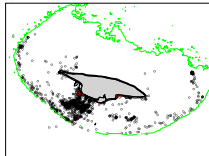

**d) Mayotte**

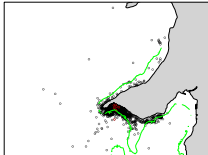

**e) La Reunion**

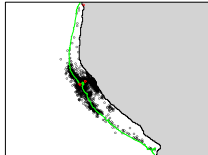

**f) Migrant turtles from Europa**

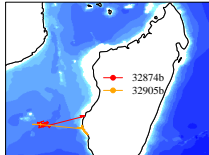

Supplement: Supplementary file 1 — Additional file 1: Figure S1. GPS locations on (a) Europa, (b) Glorieuses, (c) Juan de Nova, (d) Mayotte and (e) La Reunion. Red dots refer to release locations. (f) Migratory movements of two individuals that left Europa. [file 40462_2019_184_MOESM1_ESM.pdf]

Distance to shore (km)

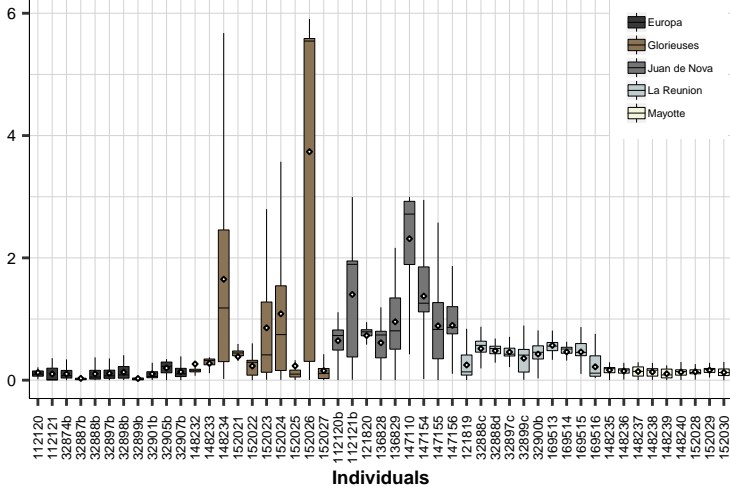

Supplement: Supplementary file 4 — Additional file 4: Figure S4. Box plots of the distance to shore extracted at each turtle location showing the inter-individual variability. [file 40462_2019_184_MOESM4_ESM.pdf]

Bathymetry (m)

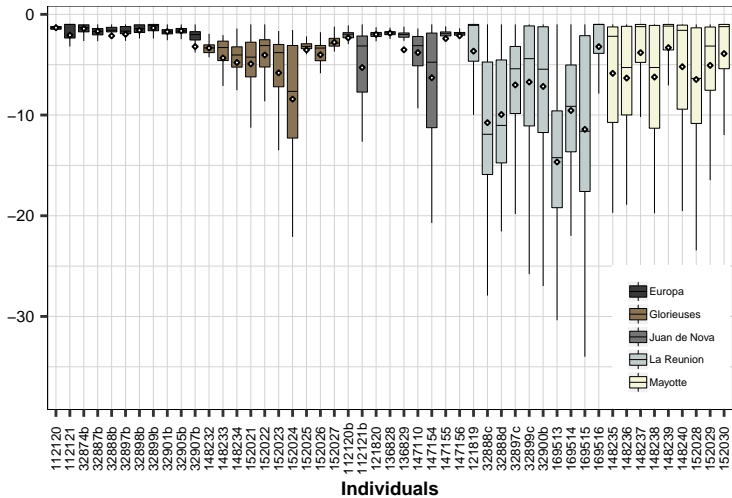

Supplement: Supplementary file 5 — Additional file 5: Figure S5. Box plots of the bathymetry extracted at each turtle location showing the inter-individual variability. [file 40462_2019_184_MOESM5_ESM.pdf]

**a) Europa**

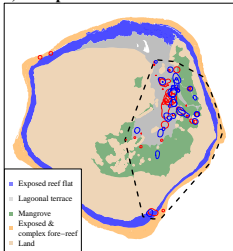

**b) Glorieuses**

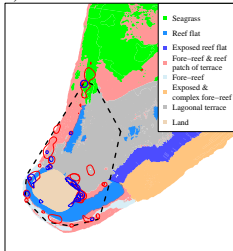

**c) Mayotte**

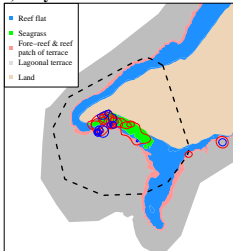

**d) La Reunion**

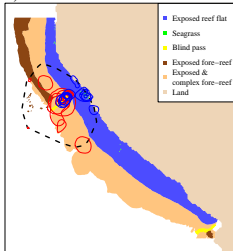

Supplement: Supplementary file 6 — Additional file 6: Figure S6. Maps of the seafloor habitats in (a) Europa, (b) Glorieuses, (c) Mayotte and (d) La Reunion. Habitat available are illustrated by the MCP (dotted black lines) and the individual habitat used by the red (diurnal) and blue (nocturnal) contours. [file 40462_2019_184_MOESM6_ESM.pdf]
