# Supplementary material for: Contrasted habitats and individual plasticity drive the fine scale movements of juvenile green turtles in coastal ecosystems
Source: Mov Ecol. 2020 Jan 7;8:1. doi: 10.1186/s40462-019-0184-2 (PMC6947949; doi:10.1186/s40462-019-0184-2)
Supplement: Supplementary file 2 — Additional file 2: Figure S2. Correlation matrices of the kernel areas tested for different tracking durations during day (left) and night (right) in (a, b) Europa, (c, d) Glorieuses, (e, f) Juan de Nova, (g, h) Mayotte and (i, j) La Reunion. Tracking durations are numbers in red. [file 40462_2019_184_MOESM2_ESM.pdf]

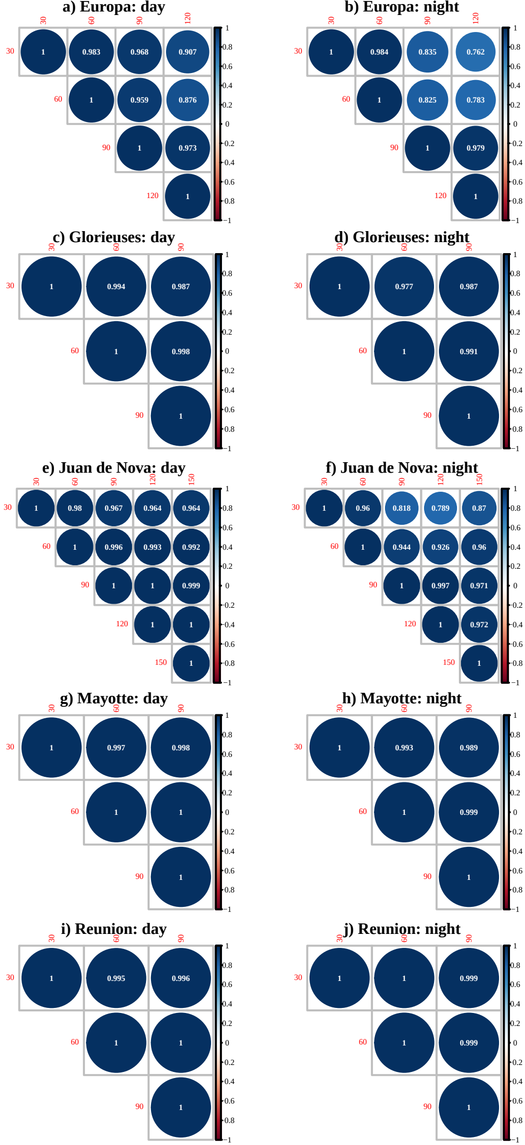

a) Europa: day
30 60 90 120
30: 1 0.983 0.968 0.907
60: 1 0.959 0.876
90: 1 0.973
120: 1
b) Europa: night
30 60 90 120
30: 1 0.984 0.835 0.762
60: 1 0.825 0.783
90: 1 0.979
120: 1
c) Glorieuses: day
30 60 90
30: 1 0.994 0.987
60: 1 0.998
90: 1
d) Glorieuses: night
30 60 90
30: 1 0.977 0.987
60: 1 0.991
90: 1
e) Juan de Nova: day
30 60 90 120 150
30: 1 0.98 0.967 0.964 0.964
60: 1 0.996 0.993 0.992
90: 1 1 0.999
120: 1 1
150: 1
f) Juan de Nova: night
30 60 90 120 150
30: 1 0.96 0.818 0.789 0.87
60: 1 0.944 0.926 0.96
90: 1 0.997 0.971
120: 1 0.972
150: 1
g) Mayotte: day
30 60 90
30: 1 0.997 0.998
60: 1 1
90: 1
h) Mayotte: night
30 60 90
30: 1 0.993 0.989
60: 1 0.999
90: 1
i) Reunion: day
30 60 90
30: 1 0.995 0.996
60: 1 1
90: 1
j) Reunion: night
30 60 90
30: 1 1 0.999
60: 1 0.999
90: 1
1 0.8 0.6 0.4 0.2 0 −0.2 −0.4 −0.6 −0.8 −1
