# Supplementary material for: Contrasted habitats and individual plasticity drive the fine scale movements of juvenile green turtles in coastal ecosystems
Source: Mov Ecol. 2020 Jan 7;8:1. doi: 10.1186/s40462-019-0184-2 (PMC6947949; doi:10.1186/s40462-019-0184-2)
Supplement: Supplementary file 3 — Additional file 3: Figure S3. Correlation matrices of the kernel areas tested for different number of locations during day (left) and night (right) in (a, b) Europa, (c, d) Glorieuses, (e, f) Juan de Nova, (g, h) Mayotte and (i, j) La Reunion. Locations numbers are numbers in red. [file 40462_2019_184_MOESM3_ESM.pdf]

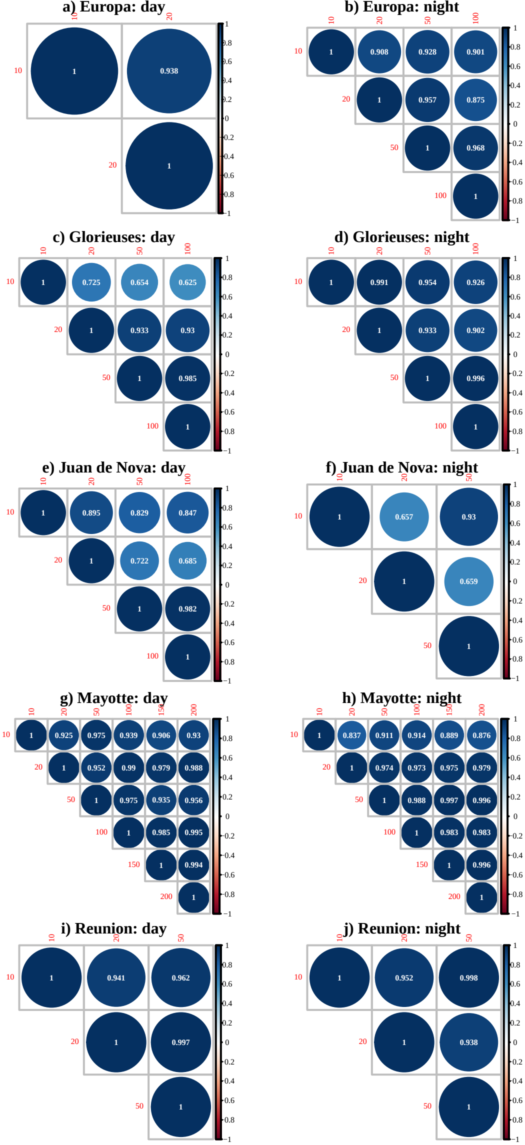
a) Europa: day
10 20
10 1 0.938
20 1
b) Europa: night
10 20 50 100
10 1 0.908 0.928 0.901
20 1 0.957 0.875
50 1 0.968
100 1
c) Glorieuses: day
10 20 50 100
10 1 0.725 0.654 0.625
20 1 0.933 0.93
50 1 0.985
100 1
d) Glorieuses: night
10 20 50 100
10 1 0.991 0.954 0.926
20 1 0.933 0.902
50 1 0.996
100 1
e) Juan de Nova: day
10 20 50 100
10 1 0.895 0.829 0.847
20 1 0.722 0.685
50 1 0.982
100 1
f) Juan de Nova: night
10 20 50
10 1 0.657 0.93
20 1 0.659
50 1
g) Mayotte: day
10 20 50 100 150 200
10 1 0.925 0.975 0.939 0.906 0.93
20 1 0.952 0.99 0.979 0.988
50 1 0.975 0.935 0.956
100 1 0.985 0.995
150 1 0.994
200 1
h) Mayotte: night
10 20 50 100 150 200
10 1 0.837 0.911 0.914 0.889 0.876
20 1 0.974 0.973 0.975 0.979
50 1 0.988 0.997 0.996
100 1 0.983 0.983
150 1 0.996
200 1
i) Reunion: day
10 20 50
10 1 0.941 0.962
20 1 0.997
50 1
j) Reunion: night
10 20 50
10 1 0.952 0.998
20 1 0.938
50 1
