# Supplementary material for: Contrasted habitats and individual plasticity drive the fine scale movements of juvenile green turtles in coastal ecosystems
Source: Mov Ecol. 2020 Jan 7;8:1. doi: 10.1186/s40462-019-0184-2 (PMC6947949; doi:10.1186/s40462-019-0184-2)
Supplement: Supplementary file 7 — Additional file 7: Table S1. Summary of the data collected from the 49 juvenile green turtles satellite tracked. N refers to the total number of GPS locations retained for the analysis. [file 40462_2019_184_MOESM7_ESM.docx]

**Table**

Table S1. Summary of the data collected from the 49 juvenile green turtles satellite tracked. N refers to the total number of GPS locations retained for the analysis.

| **Turtle ID** | **Site** | **Start date** | **End date** | **Nloc** | **Distance** | **Duration** | **Weight** | **CCL** |
| --- | --- | --- | --- | --- | --- | --- | --- | --- |
|  |  |  |  |  | **(km)** | **(d)** | **(kg)** | **(cm)** |
| 112120 | Europa | 19/11/2011 | 24/11/2011 | 13 | 5 | 5 | 17.6 | 55 |
| 112121 | Europa | 20/11/2011 | 19/01/2012 | 21 | 8 | 60 | 14 | 52 |
| 32874b | Europa | 20/11/2011 | 07/09/2012 | 73 | 71 | 292 | 30.1 | 65 |
| 32887b | Europa | 19/11/2011 | 14/03/2012 | 138 | 106 | 116 | 20.4 | 58.5 |
| 32888b | Europa | 19/11/2011 | 08/04/2012 | 224 | 180 | 141 | 15.1 | 53 |
| 32897b | Europa | 20/11/2011 | 07/04/2012 | 382 | 194 | 139 | 37.1 | 65 |
| 32898b | Europa | 20/11/2011 | 27/04/2012 | 209 | 162 | 159 | 25.7 | 62 |
| 32899b | Europa | 20/11/2011 | 18/02/2012 | 90 | 28 | 90 | 20.4 | 59 |
| 32901b | Europa | 20/11/2011 | 08/08/2013 | 85 | 74 | 627 | 26.9 | 63 |
| 32905b | Europa | 23/11/2011 | 15/12/2011 | 70 | 36 | 22 | 15.9 | 55 |
| 32907b | Europa | 25/11/2011 | 28/05/2012 | 481 | 439 | 185 | 26.5 | 63.5 |
| 148232 | Glorieuses | 02/10/2015 | 09/12/2016 | 575 | 459 | 434 | 26.25 | 61.5 |
| 148233 | Glorieuses | 28/09/2015 | 31/10/2015 | 61 | 54 | 33 | 23.1 | 59.5 |
| 148234 | Glorieuses | 30/09/2015 | 08/02/2016 | 258 | 428 | 131 | 49.1 | 75.5 |
| 152021 | Glorieuses | 28/09/2015 | 23/11/2015 | 212 | 146 | 56 | 26.1 | 63 |
| 152022 | Glorieuses | 29/09/2015 | 30/11/2015 | 310 | 212 | 62 | 19.5 | 56 |
| 152023 | Glorieuses | 26/09/2015 | 28/01/2016 | 212 | 210 | 124 | 23.4 | 61 |
| 152024 | Glorieuses | 30/09/2015 | 06/01/2016 | 104 | 91 | 98 | 12.7 | 49 |
| 152025 | Glorieuses | 30/09/2015 | 25/01/2016 | 181 | 130 | 117 | 38.8 | 71 |
| 152026 | Glorieuses | 30/09/2015 | 28/01/2016 | 216 | 223 | 120 | 20.9 | 58.5 |
| 152027 | Glorieuses | 01/10/2015 | 29/12/2015 | 202 | 146 | 89 | 12.3 | 48.5 |
| 112120b | JuandeNova | 06/04/2015 | 20/09/2015 | 170 | 168 | 167 | 15.8 | 51 |
| 112121b | JuandeNova | 06/04/2015 | 07/09/2015 | 204 | 247 | 154 | 14.4 | 51 |
| 121820 | JuandeNova | 06/04/2015 | 11/10/2015 | 140 | 146 | 188 | 11.6 | 45 |
| 136828 | JuandeNova | 06/04/2015 | 18/12/2015 | 335 | 178 | 256 | 14.8 | 50 |
| 136829 | JuandeNova | 08/04/2015 | 30/10/2015 | 294 | 121 | 205 | 22.4 | 57.5 |
| 147110 | JuandeNova | 07/04/2015 | 19/10/2015 | 91 | 77 | 195 | 22.07 | 58 |
| 147154 | JuandeNova | 07/04/2015 | 25/08/2015 | 242 | 196 | 140 | 29.8 | 53 |
| 147155 | JuandeNova | 09/04/2015 | 07/08/2015 | 251 | 258 | 120 | 19.7 | 54.5 |
| 147156 | JuandeNova | 06/04/2015 | 06/09/2015 | 293 | 157 | 153 | 13.54 | 50 |
| 148235 | Mayotte | 12/10/2015 | 11/03/2016 | 846 | 268 | 151 | 29.1 | 62.5 |
| 148236 | Mayotte | 12/10/2015 | 06/03/2016 | 501 | 253 | 146 | 41.4 | 71 |
| 148237 | Mayotte | 12/10/2015 | 07/04/2016 | 799 | 343 | 178 | 45.4 | 74 |
| 148238 | Mayotte | 12/10/2015 | 16/01/2016 | 441 | 167 | 96 | 48 | 74.5 |
| 148239 | Mayotte | 12/10/2015 | 19/12/2015 | 359 | 164 | 68 | 21.96 | 58 |
| 148240 | Mayotte | 12/10/2015 | 15/01/2016 | 580 | 248 | 95 | 46.26 | 73.5 |
| 152028 | Mayotte | 12/10/2015 | 05/03/2016 | 766 | 348 | 145 | 18.9 | 56 |
| 152029 | Mayotte | 13/10/2015 | 30/12/2015 | 558 | 296 | 78 | 35.8 | 67 |
| 152030 | Mayotte | 12/10/2015 | 08/03/2016 | 1108 | 358 | 148 | 32.1 | 66.5 |
| 121819 | Reunion | 28/11/2012 | 23/06/2013 | 937 | 214 | 207 | 12.39 | 47 |
| 32888c | Reunion | 28/11/2012 | 05/02/2013 | 1737 | 293 | 69 | 27.48 | 61.5 |
| 32888d | Reunion | 08/03/2013 | 26/03/2013 | 123 | 13 | 18 | 24.5 | 57.5 |
| 32897c | Reunion | 28/11/2012 | 31/03/2013 | 1474 | 170 | 123 | 17.64 | 53 |
| 32899c | Reunion | 28/11/2012 | 11/04/2013 | 2723 | 432 | 134 | 24.84 | 58 |
| 32900b | Reunion | 25/10/2010 | 11/02/2011 | 702 | 745 | 109 | 43 | 69 |
| 169513 | Reunion | 14/05/2018 | 01/07/2018 | 164 | 273 | 48 | 39.52 | 71 |
| 169514 | Reunion | 14/05/2018 | 28/06/2018 | 82 | 172 | 45 | 20.74 | 55 |
| 169515 | Reunion | 14/05/2018 | 29/07/2018 | 152 | 176 | 76 | 50 | 77.5 |
| 169516 | Reunion | 14/05/2018 | 28/06/2018 | 88 | 97 | 45 | 19.6 | 56 |
|  |  |  |  | *414±496* | *204±139* | *136±104* | *25.8+10.8* | *59.8±8.1* |
